# Evaluation of an eye tracking setup for studying visual attention in face-to-face conversations

**DOI:** 10.1038/s41598-021-81987-x

**Published:** 2021-01-29

**Authors:** Antonia Vehlen, Ines Spenthof, Daniel Tönsing, Markus Heinrichs, Gregor Domes

**Affiliations:** 1 Department of Biological and Clinical Psychology, University of Trier, Johanniterufer 15, 54290 Trier, Germany; 2 Department of Psychology, Laboratory for Biological and Personality Psychology, Albert-Ludwigs-University of Freiburg, Stefan-Meier-Str. 8, 79104 Freiburg, Germany

**Keywords:** Human behaviour, Behavioural methods

## Abstract

Many eye tracking studies use facial stimuli presented on a display to investigate attentional processing of social stimuli. To introduce a more realistic approach that allows interaction between two real people, we evaluated a new eye tracking setup in three independent studies in terms of data quality, short-term reliability and feasibility. Study 1 measured the robustness, precision and accuracy for calibration stimuli compared to a classical display-based setup. Study 2 used the identical measures with an independent study sample to compare the data quality for a photograph of a face (2D) and the face of the real person (3D). Study 3 evaluated data quality over the course of a real face-to-face conversation and examined the gaze behavior on the facial features of the conversation partner. Study 1 provides evidence that quality indices for the scene-based setup were comparable to those of a classical display-based setup. Average accuracy was better than 0.4° visual angle. Study 2 demonstrates that eye tracking quality is sufficient for 3D stimuli and robust against short interruptions without re-calibration. Study 3 confirms the long-term stability of tracking accuracy during a face-to-face interaction and demonstrates typical gaze patterns for facial features. Thus, the eye tracking setup presented here seems feasible for studying gaze behavior in dyadic face-to-face interactions. Eye tracking data obtained with this setup achieves an accuracy that is sufficient for investigating behavior such as eye contact in social interactions in a range of populations including clinical conditions, such as autism spectrum and social phobia.

## Introduction

Eye tracking, the video-based, non-invasive measurement of an individual’s eye movements, has become a widely used standard technique for measuring visual attention. In recent decades, numerous studies have been published that provide insights into the cause and effect of visual attention in many different contexts, such as reading^1^, consumer preferences^2^, emotion processing^3^, social interaction^4^ and psychopathology (e.g. autism: Ref.^5^). In general, attention has been investigated using well-established behavioral paradigms, such as the spatial cueing tasks^6^, the dot-probe task^7^, or classical search paradigms^8^. In addition, these basic behavioral paradigms have been extended by eye tracking to investigate the role of eye movements for attentional processes, especially in a social context^9–11^.

The majority of these studies used infrared remote eye trackers to track a participant’s eye movements while observing stimuli displayed on a screen (for review see: Ref.^12^). This technique takes advantage of the fact that stimuli that attract attention are more likely to be looked at^13^. Based on video recordings, translations of corneal reflections under infrared illumination relative to the center of the pupil are used to estimate the orientation of the eyeballs. Following appropriate calibration the gaze point on a given stimulus can be calculated^14^.

So far, many eye tracking studies in the social context have used static stimuli (e.g. facial expressions: Refs.^15,16^) or dynamic stimuli (e.g. short video clips of social interaction: Refs.^17,18^) to investigate attention to social stimuli in clinical and healthy populations. Although these studies have expanded our knowledge regarding attentional factors in the processing of social stimuli, these paradigms lack an important aspect: the possibility to interact with the social stimulus. In line with this notion, studies have reported that the physical presence^19^ and the eye contact of the counterpart^20^ significantly influences the gaze behavior of the participants.

To overcome the limitations of these so-called isolated paradigms^21,22^, eye tracking was introduced in real-life interactions with mobile eye trackers that can be worn like ordinary glasses^23,24^. Although this approach seems promising, the natural gaze behaviour could be compromised, as seeing the interaction partner wearing eye tracking glasses could lead to a constant feeling of being observed^25^.

Another approach uses network-based video communication platforms to implement standard display-based eye tracking. In this paradigm, two people communicate with each other via video streaming, while their gaze behavior is tracked on the computer display showing the interaction partner. In recent studies, this approach has been proven feasible for investigating social attention during video-based communication^26–28^. Although these approaches improve the ecological validity of eye tracking research in social situations, video-based social interactions seem to be affected by the method used for communication^29^ and the possibility of establishing eye contact is only made possible by complex technical setups^30^.

To maximize the possibilities for social interaction, at least within dyadic interactions, eye tracking systems in naturalistic setups ideally have the following characteristics^31^: low intrusiveness, flexible setup, robustness against head movement, and high accuracy. In sum, naturalistic eye tracking setups should offer sufficient data quality and at the same time give the participants the feeling of a natural conversation even in a laboratory environment.

Robustness, precision and accuracy are the main basic indices to describe the quality of eye tracking data^14^. Tracking robustness (or trackability) as the most basic parameter is calculated as the percentage of available gaze data over a predefined time period. It is determined by dividing the amount of available data by the total number of samples in that period. Data loss may be due to the fact that critical features for measuring eye movements are not detected by the eye tracker (e.g. corneal reflection or the pupil^14,32^). Precision can be defined as the average variation of the measured gaze points, where precise data leads to small deviations between individual gaze points, or in other words, compact point clouds of gaze data. Precision can be expressed as the standard deviation of the data sample (SD) or the root mean square (RMS) of inter-sample distances as recommended in the literature^14^. Accuracy can be referred to as the trueness of gaze measurement and can be defined as the average mean offset between the measured gaze position and the actual target point on the stimulus display, or in other words, data is accurate when the distance between the centroid of the measured data and the target point is small.

Under standard laboratory conditions (constant distances, luminance, restriction of head movements, etc.), most eye trackers offer excellent robustness of recording, with only minimal proportions of lost data, at least from the majority of participants. Precision can be increased to a certain degree by using low-pass filtering or smoothing of the raw data. Therefore, accuracy can be considered the most crucial and problematic parameter in the majority of studies.

Eye tracker manufacturers usually specify accuracy and precision of their products after testing them under ideal conditions, e.g. display-based setup with the optional usage of a chinrest depending on the product. As an example, for a standard remote infrared eye tracker (e.g. Tobii X3-120), accuracy and precision (SD) has been reported in the product description of the manufacturer with values of 0.4 and 0.47°, respectively^33^. A visual angle of 0.4° is equivalent to 4 mm on a computer display at a standard viewing distance of 65 cm. In other words, fixations on two stimuli with a distance of more than 4 mm can be reliably distinguished at a viewing distance of 65 cm.

However, it has been shown that eye trackers typically show lower accuracy and precision when applied under non ideal conditions (e.g. absence of chinrest^34^). For example the Tobii X2-30 was specified with an accuracy of 0.4° and precision (SD) of 0.26°^35^, but an independent evaluation estimated an accuracy and precision (SD) of 2.5 and 1.9°, respectively^36^. This is problematic, since a deviation in accuracy even as small as 0.5° has been shown to significantly change results regarding the gazing durations for multiple areas of interest (AOIs)^37^. The problem becomes even greater with increasing viewing distance, as is the case in face-to-face conversations. With increasing viewing distance, the spatial resolution on the display plane decreases depending on the level of accuracy. A viewing distance of 150 cm at an accuracy of 0.5° would lead to a spatial resolution of approximately 1.5 cm. Therefore, it is important to evaluate experimental setups in the field of eye tracking research with respect to the research questions, taking into account data accuracy as one of the limiting factors when observing gaze behavior on small areas of interest such as the eye region.

In the following, we introduce an eye tracking setup that has been developed to track the gaze behavior of one participant in a dyadic interaction. The use of a remote eye tracker placed at the rim of the field of view conceals the fact that the gaze is being recorded while maintaining a maximum of freedom in head and body movement. The aim is to achieve high quality eye tracking data without limiting the natural feel of the examined situation. In three independent studies we measured robustness, precision and accuracy of this setup, compared it to a standard display-based setup, tested its feasibility to measure gaze behavior on 3D stimuli, examined the effect of short interruptions and observed data quality and gaze pattern on another person during a face-to-face conversation.

## Study 1: Comparison of display-based and scene-based eye tracking

In the first study, we compared an infrared remote eye tracker’s robustness, precision and accuracy in a standard computer display-based setup with the scene-based setup. This scene-based setup can be used to assess gaze behavior for stimuli that are not presented on a computer display but rather in naturalistic environment, such as a conversation partner in social interactions or any other face-to-face scenario. The display-based setup served as a control condition for eye tracking under ideal conditions.

### Methods

#### Participants

Twenty participants were recruited via on-campus announcements at the University of Trier. The exclusion criteria covered visual impairments exceeding ± 0.5 diopters and any type of prescriptive visual aid. Upon arrival at the laboratory, participants completed an online questionnaire to monitor the exclusion criteria and other factors that affect the quality of eye tracking data such as eye infections or allergic reaction leading to watery or irritated eyes. No participants had to be excluded. The final sample comprised of 20 healthy participants (10 male/10 female) aged 26 ± 4 years. The ethics committee at the University of Trier approved the study. The research was conducted in accordance to the Declaration of Helsinki. All participants gave written informed consent and received 10 € after completion of the study. Individuals who are identifiable in the images and video gave informed consent for publication.

#### Setup and stimuli

In both conditions, data was recorded at a sampling frequency of 120 Hz with a Tobii X3-120 remote infrared eye tracker. Both setups were placed in the same bright room with constant lighting and temperature. Constant lighting was assured by blocking daylight by means of window blinds and using diffuse artificial lighting attached to the ceiling.

##### Display condition

In the standard display-based setup, the eye tracker was attached to a standard 24 in. monitor (16:9) with a 1920 px × 1080 px resolution connected to a standard PC running a Nvidia Quadro P620 graphics card and Windows 10. The eye tracker was attached via mounting brackets to the bottom of the monitor and tilted by an angle of 25°. Participants were placed at a distance of 65 cm in front of the eye tracker. Eye movement data were recorded with a script written in Python (version 2.7) using the API of Tobii Pro SDK (http://developer.tobiipro.com/python.html). The calibration stimuli were black points on a white background with an initial radius of 1.51 cm (1.5°; see Fig. 2a for an image of the calibration area). The points were horizontally 22 cm (20.5°) and vertically 12.4 cm (11.9°) apart, resulting in a calibration area of 44.0 × 24.8 cm (active display size 53 × 30 cm; 48.4 × 28.5°; see Fig. 2a). Once the stimulus appeared, the participants had 500 ms to fixate on it. After this period, the stimulus shrank continuously in size to a radius of 0.14 cm.

##### Scene condition

**Figure 1:**
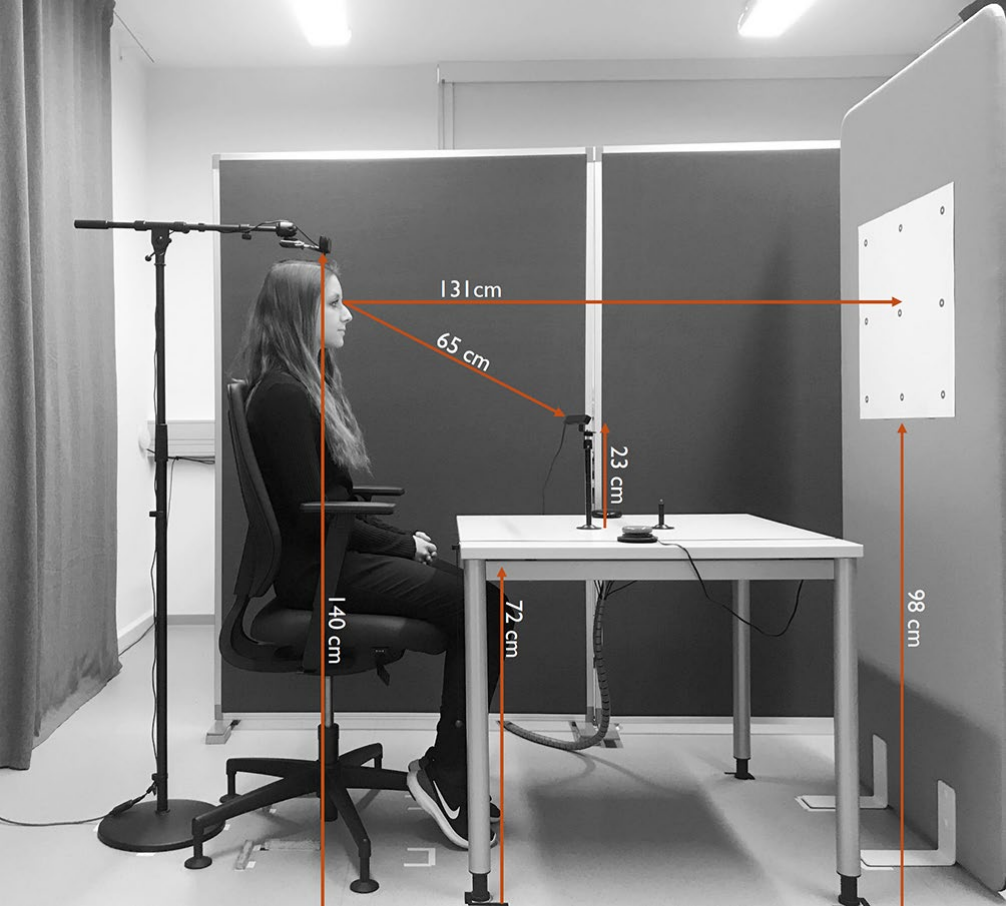
Illustration of the scene setup with relevant distances.

**Figure 2:**
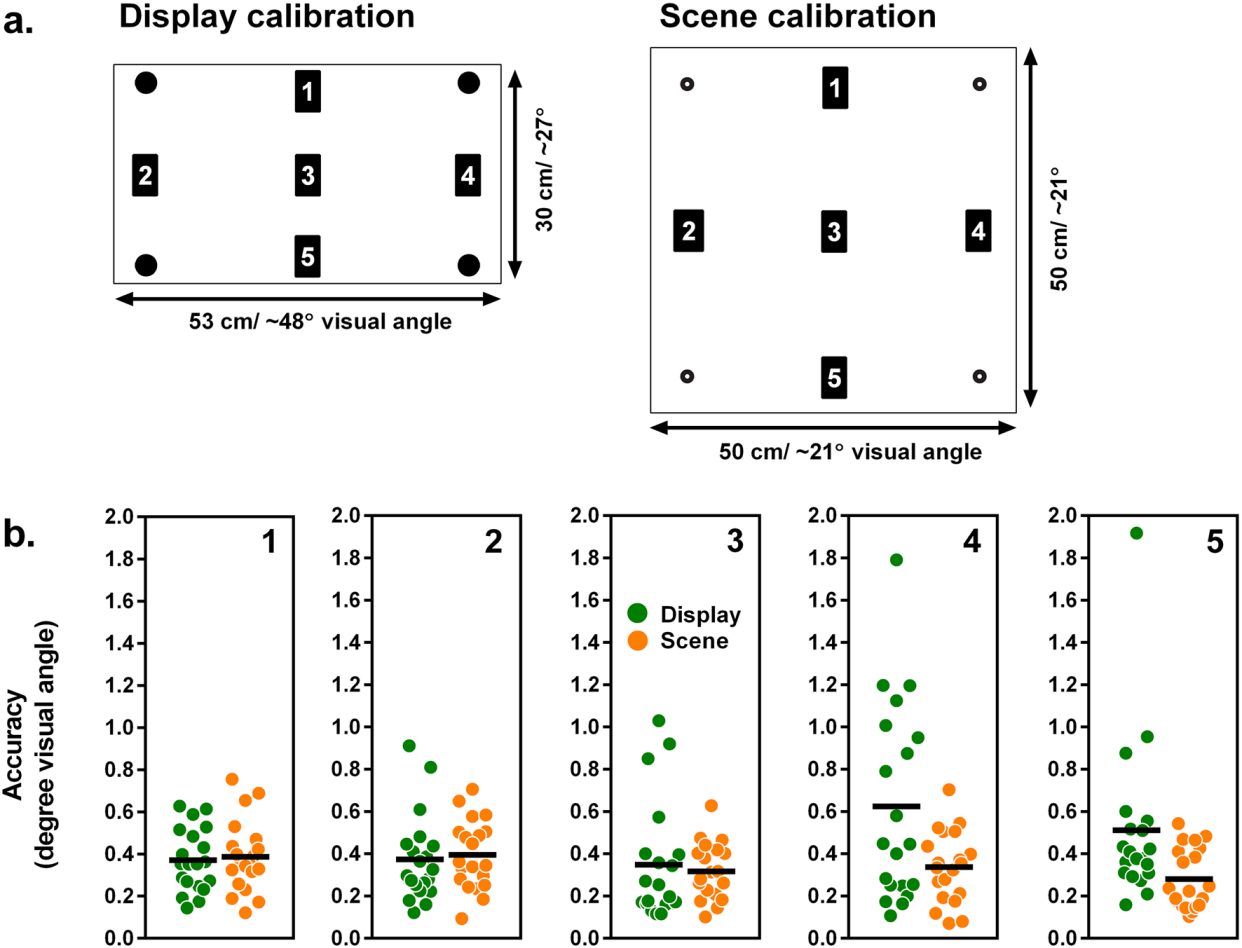
Accuracy for display and scene condition at different target locations. (**a**) Size and target location for display and scene calibration. (**b**) Accuracy in degrees visual angle. Black lines represent mean values. Lower values represent better accuracy.

The eye tracker was placed on a table (80 × 80 cm; height: 72 cm) using a fixed monopod (height: approx. 23 cm). The eye tracker was tilted at an angle of 28° and connected to an external processing unit fixated under the table. This unit was connected to a standard PC running Tobii Studio Pro (version 3.4.5) to record eye tracking data and the corresponding video stream. A webcam (Logitech c920) was placed at a height of 140 cm for recording the scene from the participant’s point of view (see Fig. 1) with a 1920 px × 1080 px resolution. Participants sat on a height-adjustable chair in front of the eye tracker at a distance of 65 cm. Before the experiment started, the chair was individually adjusted in height so that each participant sat directly under the camera. The setup with the corresponding distances is shown in Fig. 1. The calibration stimuli were black circles on a white sheet with a radius of 1.45 cm (~ 1.3°). The points were 20 cm (~ 8.7°) vertically and horizontally apart, resulting in a calibration area of 40 × 40 cm (active display size 50 × 50 cm; 21.6 × 21.6° visual angle; see Fig. 2a). The calibration sheet was attached to a light grey vertical partition wall (180 × 80 cm) that could be moved after the calibration procedure was completed.

#### Design and procedure

The study used a within-subject design to directly compare tracking robustness, precision and accuracy for presentation without a computer display (scene condition) and a standard computer display-based setup (display condition). To control for sequence effects, the order of the conditions was randomized.

##### Display condition

The condition started with a 9-point-calibration procedure, followed by three 5-point-validation sequences with points at different fixation locations across the calibration area (see Fig. 2a). During the calibration sequence, the points appeared automatically on the screen, and at the end of the sequence the experimenter received numerical feedback on the quality of the calibration. The calibration sequence was repeated up to three times if calibration quality exceeded an accuracy of 1°. Once the calibration was successfully completed, the three validation sequences followed, in which five of the calibrations points were shown in randomized order, resulting in 15 trials per condition per participant. The top center (1), the three points from the middle row (2–4) and the bottom center (5) of the calibration points were chosen as target locations to represent the usual presentation area of stimuli. The points automatically reappeared on the screen, and the program set the markers used for the offline segmentation of the eye tracking data accordingly.

##### Scene condition

The calibration and validation sequence was adapted for the scene condition. The nine target points of the calibration sequence were printed on a poster and announced by the experimenter. The feedback regarding calibration quality was not automatically quantified by the Tobii software, but provided in form of a visual display which was quantified by an in-house AutoIt script. Again, calibration sequences were repeated up to three times if calibration quality exceeded an accuracy of 1°. The procedure of the following validation sequences differed from the display condition in that the points were announced verbally by the experimenter in 2-s intervals, i.e. the experimenter announced one point and pressed a marker as soon as the participant looked at the corresponding target (visible in a live video provided by the Tobii software Pro Studio) and then waited 2 secs before announcing the next target.

#### Data analyses

##### Preprocessing

All calculations were based on averaged binocular data. Segmented raw gaze data was averaged over the three trials per participant. Every single segment comprised of 120 data points (1 s). The raw data of the segments were visually examined for outliers from the centroid by plotting individual gaze data on the corresponding calibration sheet. Of the 600 trials, start markers of 12 trials (2%) had to be corrected to exclude outlier data points. One trial had to be excluded due to experimenter error (0.17%).

##### Calculation of robustness, precision, and accuracy

Robustness was calculated as the percentage of available data, i.e. by dividing the number of valid data points (as coded in the data output of the Tobii software) by the total number of data points and multiplying by one hundred. Precision (1) as a measure of variance was calculated via the standard deviation (SD) of data samples and is reported in degrees of visual angle and in centimeter accordingly. Again, low values correspond to high precision.

1 $$\theta_{SD} = \sqrt {\frac{1}{n}\sum\limits_{i = 1}^{n} {\mathop {\left( {x_i - \overline{x}} \right)}\nolimits^{2} } + \frac{1}{n}\sum\limits_{i = 1}^{n} {\mathop {\left( {y_i - \overline{y}} \right)}\nolimits^{2} } }$$

Accuracy (2) was calculated as the mean offset of the gaze point position in relation to the target point position and is reported in degrees of visual angle and in centimeter. Thus, low values correspond to high accuracy.

2 $$\theta_{offset} = \sqrt {\mathop {\left( {x_{target} - \overline{x} \, } \right)}\nolimits^{2} + \mathop {\left( {y_{target} - \overline{y}} \right)}\nolimits^{2} } \,$$

Calculations of basic quality indices were conducted via in-house scripts in Matlab (2019, version 9.7.0).

##### Statistical analyses

The influence of the within-subject factors condition (display vs. scene) and target location (target locations 1–5 of the validation sequences) were tested for robustness, precision, and accuracy with repeated measures ANOVAs. Follow-up repeated measures ANOVAs with the factor target location and the respective post-hoc comparisons were conducted for each condition separately. Post-hoc single comparisons are reported with Bonferroni correction. In cases of heterogeneity of covariance (Mauchly test of sphericity) Greenhouse–Geisser corrections were applied. All statistical analyses were done in R version 4.0.0^38^ with the level of significance set to *p* < 0.05. Effect sizes are reported as ƞ^2^_G_ to ensure comparability with other studies^39^.

### Results

#### Effects of condition and target location on robustness, precision and accuracy

The 2 (condition) × 5 (target location) ANOVA for robustness revealed a significant main effect of condition, *F*(1,19) = 45.38, *p* < 0.001, ƞ^2^_G_ = 0.32, but neither a significant main effect of target location, *F*(2.98,56.64) = 0.79, ε = 0.75, *p* = 0.502 , ƞ^2^_G_ = 0.01, nor a significant interaction effect, *F*(2.99,56.79) = 0.89, ε = 0.75, *p* = 0.454, ƞ^2^_G_ = 0.01. The robustness was better in the scene compared to the display condition (see Table 1).

**Table 1:** Eye tracking robustness, precision and accuracy under standard laboratory conditions for a standard display setup (display condition) and the scene-based setup (scene condition). Values in degrees of visual angle and cm.

	Display				Scene			
	Min	Max	Mean	SD	Min	Max	Mean	SD
**Robustness**								
% valid data	77.56	96.39	89.55	6.64	94.61	100	98.75	1.66
**Precision (SD)**								
In degrees	0.33	0.60	0.41	0.07	0.20	0.53	0.34	0.10
In cm	0.33	0.61	0.42	0.07	0.46	1.21	0.78	0.23
**Accuracy**								
In degrees	0.17	1.01	0.45	0.20	0.18	0.47	0.34	0.08
In cm	0.18	1.04	0.46	0.21	0.41	1.07	0.78	0.18

Precision (SD) = standard deviation of data samples (Holmqvist et al., 2011).

The ANOVA for precision (SD) with the within-subject factors condition and target location showed a significant main effect of condition, *F*(1,19) = 7.79, *p* = 0.012, ƞ^2^_G_ = 0.08, a significant main effect of target location, *F*(2.54,48.22) = 4.77, ε = 0.63, *p* = 0.008, ƞ^2^_G_ = 0.08, and a significant interaction effect, *F*(2.62,49.79) = 11.11, ε = 0.66, *p* < 0.001, ƞ^2^_G_ = 0.14. Descriptive data show that precision of data was better in the scene condition compared to the display condition (see Table 1).

The follow-up repeated measures ANOVA revealed a significant main effect of target location in the display condition, *F*(1.70,32.31) = 10.43, ε = 0.43, *p* < 0.001, ƞ^2^_G_ = 0.29, as well as in the scene condition, *F*(3.21,61.03) = 3.68, ε = 0.80, *p* = 0.015, ƞ^2^_G_ = 0.09. Post-hoc single comparisons in the display condition showed that the following points differed significantly in precision: 1 vs. 5, *t*(76) = − 6.18, *p* < 0.001, *r* = 0.58, 2 vs. 5, *t*(76) = − 4.70, *p* < 0.001, *r* = 0.48, 3 vs. 5, *t*(76) = − 3.41, *p* = 0.011, *r* = 0.34) and 4 vs. 5, *t*(76) = − 3.44, *p* = 0.009, *r* = 0.37, with reduced precision at point 5. The post-hoc comparisons for the scene condition revealed a significant difference between location 2 vs. 5, *t*(76) = 3.02, *p* = 0.034, *r* = 0.33 and location 4 vs. 5, *t*(76) = 3.12, *p* = 0.026, *r* = 0.34, with better precision at location 5.

The same ANOVA for accuracy revealed no significant main effect of condition, *F*(1,19) = 3.71, *p* = 0.069, ƞ^2^_G_ = 0.04, no significant effect of target location, *F*(3.15,59.81) = 2.45, ε = 0.79, *p* = 0.070, ƞ^2^_G_ = 0.04, but a significant interaction of condition and target location, *F*(2.51,47.63) = 4.15, ε = 0.63, *p* = 0.015, ƞ^2^_G_ = 0.07, indicating that target location differentially influenced tracking accuracy in the display and scene condition. Accordingly, the follow-up repeated measures ANOVAs revealed that in the display condition tracking accuracy differed between the target locations, *F*(2.88,54.70) = 3.74, ε = 0.72, *p* = 0.018, ƞ^2^_G_ = 0.11. Accuracy values for each target location in the display condition can be found in Fig. 2b. Post-hoc comparison for the display condition revealed significant differences between locations 1 vs. 4, *t*(76) = − 2.91, *p* = 0.047, *r* = 0.24 and locations 3 vs. 4, *t*(76) = − 3.17, *p* = 0.022, *r* = 0.34, with better accuracy at locations 1 and 3. In contrast, in the scene condition tracking accuracy was equal for all target locations, *F*(3.22,61.21) = 2.00, ε = 0.81, *p* = 0.119, ƞ^2^_G_ = 0.07.

## Discussion

In sum, the results of the first study showed that both display-based and scene-based eye tracking is possible with comparable robustness, precision and accuracy. Using a standard display-based setup, we were able to reproduce previously reported values of precision and accuracy for the specific eye tracker used in the present study. In the scene-based setup tracking for a 2D stimulus, average accuracy is approx. 0.34° which corresponds to approx. 8 mm at a viewing distance of 131 cm. Thus, using the specific eye tracker (in this study a Tobii X3-120) in a scene-based setup can provide data that allows distinguishing gaze points that are at least 1.6 cm apart, taking into account the imprecision of the eye tracker. This accuracy seems to be sufficient for most studies in the context of social interactions, as it enables a valid distinction to be made for fixations on common regions of interest, such as the eyes and the mouth or other facial features.

However, accuracy obviously depends on the location of the target. The central location showed the best data quality in the display condition, while data quality declined towards the edges of the calibration area with increasing visual angle. This is in line with previous reports (e.g. Ref.^32^) and indicates that stimuli should be ideally placed in the center of the calibration area. However, the present results suggest that in the scene condition tracking quality, especially accuracy, is less dependent on stimulus location, as there were no significant differences in accuracy for the different target locations during validation. This might be mainly due to the lower visual angle covered by the calibration area in the scene condition, compared to the display condition.

## Study 2: Data quality for 2D and 3D and reliability of calibration

In the second study we aimed to test eye tracking data quality of the setup for 3D compared to 2D stimuli and the reliability of eye tracking quality by dividing the study into two runs without re-calibration in between. In addition, we used the same validation procedure as in study 1 in order to replicate the findings regarding basic quality indices.

### Methods

#### Participants

An independent sample of twenty-two participants were recruited for this study in the same way as in the first study. All exclusion criteria were identical to the first study. The online questionnaire again served to check the exclusion criteria and led to the exclusion of two potential participants due to visual impairment over or below the specified threshold (*n* = 2). The final sample consisted of 20 healthy participants (10 male/10 female) with a mean age of 24 ± 3 years. Written informed consent was given by all participants in exchange for 0.5 course credits or 5 € on completion of the study. The research was conducted in accordance to the Declaration of Helsinki and the ethics committee at the University of Trier approved the study. Individuals who are identifiable in the images and video gave informed consent for publication.

#### Setup and stimuli

The second study took place in the same eye tracking laboratory as the first study using only the scene-based setup. All settings as well as the calibration procedure were identical to the first study to provide the possibility of replicating the results. In this study, data were recorded on calibration stimuli, identical to study 1. Additionally, we recorded data on another stimulus, namely the picture of a male person (2D condition), and the person himself (3D condition).

##### 2D vs. 3D stimulus

**Figure 3:**
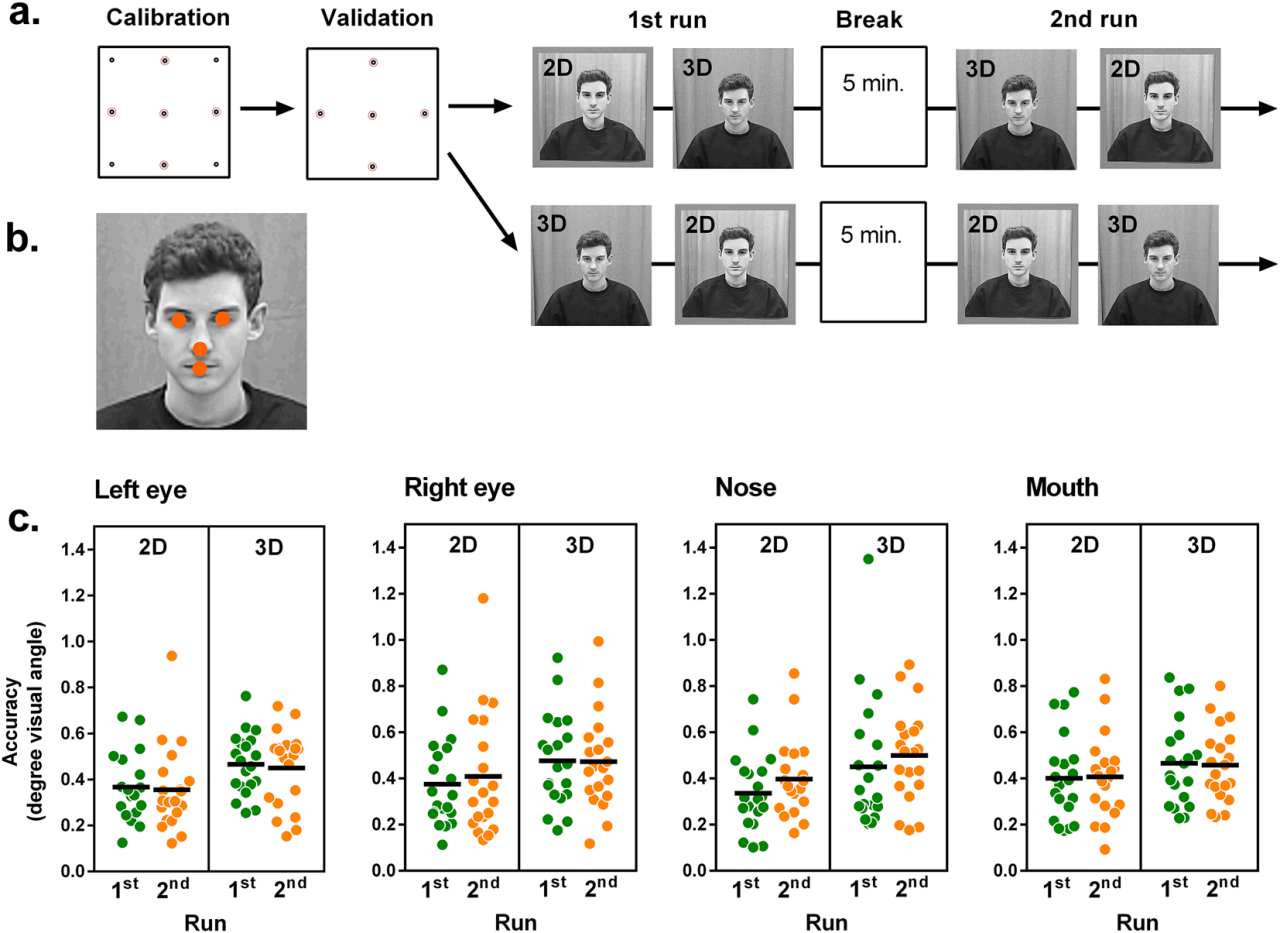
Accuracy for a 2D and a 3D stimulus. (**a**) In a cross-over design half of the sample started with 2D stimulus. During the 5 min break participants stood up and left the room. (**b**) Participants were instructed to fixate on the eyes, nose, and mouth. (**c**) Accuracy for the different facial landmarks in degrees visual angle. Black lines represent mean values. Lower values represent better accuracy.

For the life-sized 2D stimulus, a photograph of a male person was taken. The edited picture was printed on a 50 × 50 cm poster resembling the active display area of the scene condition. The face covered a visual angle of 7.8 × 5.5° or 17.9 × 12.6 cm, respectively. The poster was attached on the same partition wall as the calibration stimuli, in the position where an actual person would sit. For the 3D condition, the vertical partition wall was replaced by a chair and the male person depicted on the poster took a seat. All visual characteristics (background, clothing, etc.) were kept constant between the poster and real person (see Fig. 3a).

#### Design and procedure

The session started with the calibration procedure (as described in the “Methods” section of study 1). After calibration, the three recordings of the 5-point-validation of study 1 were repeated to replicate the results of study 1 with an independent sample of participants.

Next, eye tracking quality was tested for facial stimuli in a 2 (2D vs. 3D) × 2 (1st vs. 2nd run) × 4 (facial features) within-subject design. In randomized order, this part of the study started with either the 2D or 3D condition (see Fig. 3a). In both conditions three repetitions were conducted comprising of the following target locations: left eye, right eye, nose and mouth (see Fig. 3b), resulting in 12 trials per condition per participant. After the participants had completed this procedure, they were asked to leave the room and take a seat in a waiting area outside the laboratory. After 5 min the participant returned to their seat in the laboratory and in reversed order another three recordings of both conditions followed (see Fig. 3a for the study procedure).

#### Data analyses

##### Preprocessing

Averaged binocular gaze data was again used for all subsequent calculations. We applied the same procedure as in study 1. Individual trials were inspected for outliers and corrected when required. Of the total 1260 trials, markers of 41 trials (3.25%) had to be corrected due to outliers. One trial had to be excluded due to experimenter error (0.08%).

##### Calculation of robustness, precision, and accuracy

Parameters of the validation sequence on the calibration sheet were calculated in accordance with study 1. The calculations for the 3D conditions differed slightly due to potential movement of the stimulus. In order to acquire the exact position of the target points for every frame of recorded data, the facial landmark detection tool OpenFace^40^ was used. The four target locations (center of left and right pupil, nose tip and middle of the upper lip) were computed from the detected landmarks. For comparability of the 2D and 3D condition, OpenFace was also used for the 2D stimulus, even though movement was not possible.

##### Statistical analyses

The quality indices of the scene-based conditions were compared between study 1 and study 2 by means of independent *t*-tests. The influence of the third-dimension, the reliability of eye tracking and the effect of target location was evaluated with three-factorial analysis of variance (ANOVA) with the within-subject factors condition (2D vs. 3D), time (1st run vs. 2nd run) and target location (left eye, right eye, nose, and mouth). In case of heterogeneity of covariance (Mauchly test of sphericity) Greenhouse–Geisser corrections were applied. All statistical analyses were conducted in R with the level of significance set to *p* < 0.05. Effect sizes are reported as Pearson correlation coefficient *r* for *t*-tests and generalized eta squared ƞ^2^_G_ for ANOVAs.

### Results

#### Robustness, precision and accuracy

Comparing robustness, precision and accuracy for the independent samples of the two studies revealed the following results: There were significant differences in accuracy: *t*(38) = 2.05, *p* = 0.047, *r* = 0.32 and robustness: *t*(38) = 2.48, *p* = 0.018, *r* = 0.37, but not in precision (SD): *t*(38) = 0.47, *p* = 0.642, *r* = 0.08. The statistical significance was brought about by slightly better quality indices in study 2 compared to study 1 regarding accuracy: *M* = 0.29°, *SD* = 0.08 vs. *M* = 0.34°, *SD* = 0.08 and an opposing trend regarding robustness: *M* = 97.17%, *SD* = 2.32 vs. *M* = 98.75%, *SD* = 1.66. Precision (SD) differed between the studies as follows: *M* = 0.32°, *SD* = 0.08 (study 1) vs. *M* = 0.34°, *SD* = 0.10 (study 2).

#### Reliability of eye tracking quality on facial features of a 2D and 3D stimulus

The ANOVA on robustness with the within-subject factors condition, time and target location revealed no significant main effect of condition, *F*(1,19) = 3.87, *p* = 0.064, η^2^_G_ = 0.03, no significant main effect of time, *F*(1,19) = 1.81, *p* = 0.195, η^2^_G_ = 0.01, and no significant main effect of target location, *F*(2.09,39.62) = 0.50, ε = 0.70, *p* = 0.615, η^2^_G_ < 0.01. The two-way interaction effects between condition and time, *F*(1,19) = 0.03, *p* = 0.863, η^2^_G_ < 0.01, and condition and target location, *F*(2.34,44.55) = 0.32, ε = 0.78, *p* = 0.758, η^2^_G_ < 0.01, were not significant either. However, the interaction of time × target location, *F*(2.54,48.30) = 3.40, ε = 0.85, *p* = 0.031, η^2^_G_ = 0.01, and the three-way interaction, *F*(2.53,47.98) = 2.98, ε = 0.84, *p* = 0.049, η^2^_G_ = 0.01 were significant.

Regarding precision (SD), the ANOVA with the within-subject factors condition, time and target location revealed a significant main effect of condition, *F*(1,19) = 7.82, *p* = 0.012, η^2^_G_ = 0.07, but no significant main effect of time, *F*(1,19) = 0.04, *p* = 0.847, η^2^_G_ < 0.01, no significant main effect of target location, *F*(2.75,52.22) = 0.58, ε = 0.92, *p* = 0.616, η^2^_G_ < 0.01 and no significant two-way interactions of condition × time, *F*(1,19) = 0.50, *p* = 0.488, η^2^_G_ < 0.01, and of condition × target location, *F*(2.56,48.73) = 0.11, ε = 0.85, *p* = 0.936, η^2^_G_ < 0.01. The time × target location interaction, as well as the three-way interaction were significant, *F*(2.24,42.53) = 3.95, ε = 0.86, *p* = 0.023, η^2^_G_ = 0.02 and *F*(2.77,52.72) = 2.89, ε = 0.93, *p* = 0.048, η^2^_G_ = 0.02.

Finally, the ANOVA on accuracy with the within-subject factors condition, time and target location again exposed a significant main effect of condition, *F*(1,19) = 8.18, *p* = 0.010, η^2^_G_ = 0.08, with a slightly higher accuracy in the 2D condition (see Table 2).

**Table 2:** Robustness, precision, accuracy for eye tracking on a 2D and 3D face stimulus before and after a 5-minute break. Quality indices were averaged for fixations on eyes, nose, and mouth.

	2D				3D			
	1st run		2nd run		1st run		2nd run	
	Mean	SD	Mean	SD	Mean	SD	Mean	SD
**Robustness**								
% valid data	97.39	2.74	98.09	1.68	96.02	4.86	96.89	2.48
**Precision (SD)**								
In degrees	0.38	0.10	0.37	0.08	0.43	0.08	0.43	0.12
In cm	0.87	0.23	0.85	0.18	0.98	0.18	0.98	0.27
**Accuracy**								
In degrees	0.37	0.13	0.39	0.16	0.46	0.17	0.47	0.14
In cm	0.85	0.30	0.89	0.37	1.05	0.39	1.07	0.32

Precision (SD) = standard deviation of data samples (Holmqvist et al., 2011).

**Figure 4:**
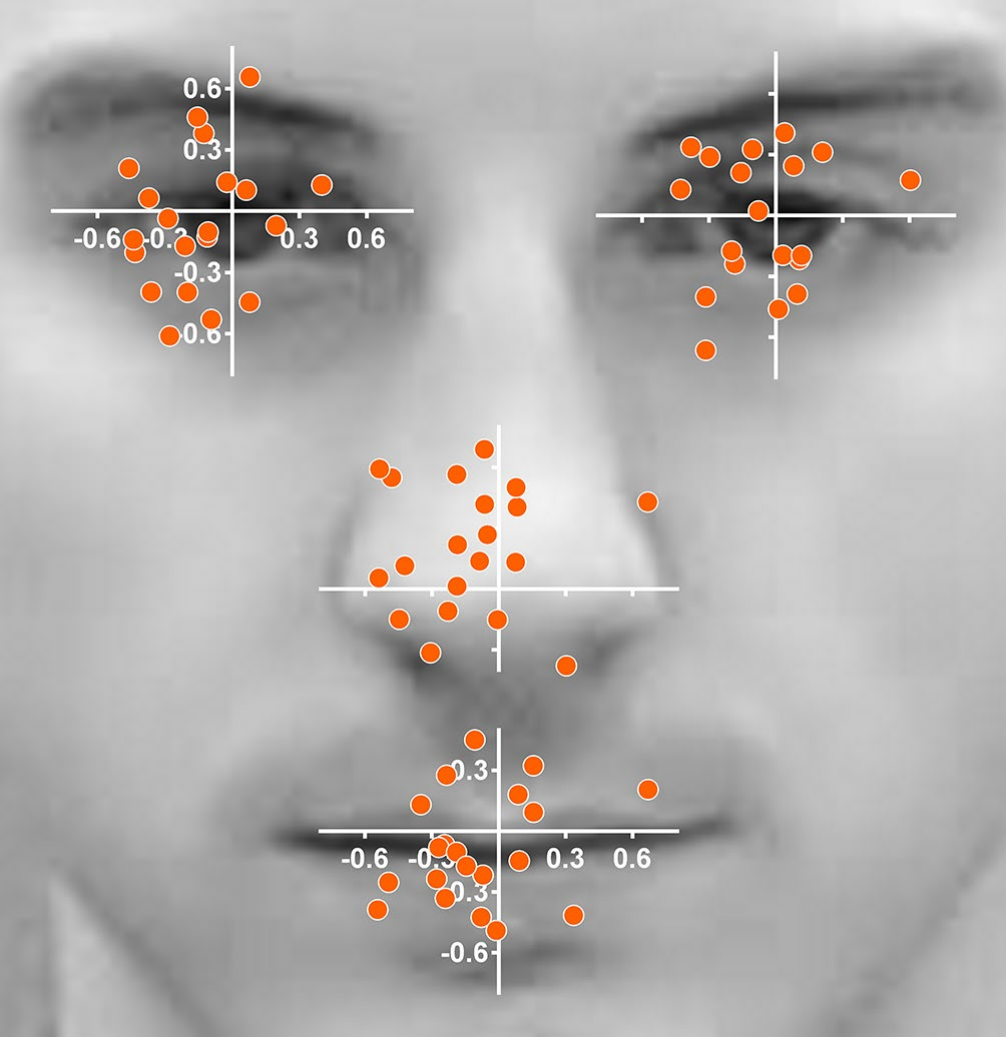
Illustration of individual accuracy for fixations on the eyes, nose and mouth. Data is presented for the 3D stimulus and the second run. Dots represent the average gaze points of a single participant over three repetitions fixating at a specific target (see “Methods” for details). The scale represents degrees visual angle.

The other main effects were not significant (time: *F*(1,19) = 0.47, *p* = 0.504 , η^2^_G_ < 0.01, target location: *F*(2.05,38.97) = 0.28, ε = 0.68, *p* = 0.763, η^2^_G_ < 0.01). The same held true for all interaction effects (condition × time: *F*(1,19) = 0.11, p = 0.739 , η^2^_G_ < 0.01, condition × target location: *F*(2.42,45.95) = 0.48, ε = 0.81, *p* = 0.660, η^2^_G_ < 0.01, time × target location: *F*(2.85,54.06) = 1.39, ε = 0.95, *p* = 0.256, η^2^_G_ < 0.01 and condition × time × target location: *F*(2.38,45.20) = 0.13, ε = 0.79, *p* = 0.908, η^2^_G_ < 0.01) (see Fig. 3c). Thus, accuracy was slightly better for the 2D compared to 3D stimulus, but stable over the course of the study and independent of the facial feature the participants were fixating on. An illustration of the accuracy for the facial features is given for the 2nd run of the 3D condition in Fig. 4.

### Discussion

In sum, the results of study 2 show that remote eye tracking used in this scene-based setup can be used to track fixations for facial features with high reliability and validity. First, we were able to achieve comparable data quality as in study 1. Second, we were able to show that the quality of eye tracking is comparable for 2D and 3D stimuli placed at a distance from the participants that is common for dyadic conversations. Third, once calibrated, eye tracking quality is stable even if the eye to tracker connection is interrupted for a few minutes and participants are reseated without re-calibration.

The present study demonstrates that when applying the scene-based setup as described above, remote eye tracking is suitable for detecting fixations on different facial features such as the eyes or the mouth with high accuracy and thus validity. Accuracy of 3D stimuli (such as a real person in the present study) is slightly lower compared to a 2D representation. This is most likely due to the parallax error occurring when eye tracking camera and scene camera are not co-axial. This error, usually eliminated by calibration on a static plane with a defined distance, negatively affects eye tracking accuracy when the observed stimulus moves along the z-axis away from the calibration plane^41^. Although the observed person, i.e. the stimulus in the present study, was instructed to sit still and refrain from large movements (especially on the z-axis), head movements cannot be completely ruled out. Related to this issue, although no differences between the facial features differing along the z-dimension (e.g. eyes and nose tip) were found in this study, a larger spatial deviation of the target points from the calibration sheet layer could significantly affect accuracy and should therefore be systematically investigated in future studies.

Furthermore, the study provides evidence that the calibration is stable, even if eye tracking is interrupted and the participant sits down again after a short break. This finding may seem surprising, but becomes clearer when one considers that the chair was not moved and participants were instructed to return to their former seating position. Although we have not tested the effect of head position on calibration stability in our setup specifically, other studies using remote eye trackers have already shown that data quality depends on the head’s position within the tracking area^42^. In our study, the webcams over the heads of the participants might have served as a reference point for the experimenter to evaluate and adjust the head position and thus might explain the good data accuracy even after the break.

## Study 3: Data quality and gaze behavior in a face-to-face conversation

The third study was conducted to explore the feasibility of the setup and to assess data quality in a standardized face-to-face conversation in which head movements and facial expressions naturalistically occur. Therefore, the robustness, precision and accuracy were calculated for four facial features before and after the conversation. In addition, data quality during the conversation was approximated based on fixations to the corresponding AOIs in speaking as well as listening phases. Furthermore, a movement index was calculated to estimate head movements and facial expression of the conversation partner during the conversation and to assess the relationship between these movements and data quality. Finally, total dwell time on AOIs covering eyes, nose, mouth, the face as whole as well as surrounding areas and the proportion of missing data was calculated during an actual dyadic conversation.

### Methods

#### Participants

A sample of 51 participants was recruited in the same manner as the other two studies. Exclusion criteria were visual impairments exceeding ± 1.5 diopters, any type of prescriptive visual aid other than soft contact lenses, acute eye inflammation and prior eye surgery. Since we wanted to investigate dynamic gaze behavior in a non-clinical sample, psychotherapeutic treatment in the last 2 years, neurological diseases, and residual symptoms of a brain injury or the intake of psychotropic drugs also led to the exclusion of participants. Because all participants interacted with a female research assistant (RA) we chose an all-female sample to minimize effects of attraction on gaze behavior. Six participants were excluded from analysis because of technical problems during the testing session (*n* = 6). Other participants retrospectively reported watery eyes (*n* = 1), visual impairment above the predefined threshold (*n* = 1) and astigmatism (*n* = 1) during the session, which also led to exclusion. The final sample consisted of 42 participants with a mean age of 23 ± 3 years. The study was conducted in accordance to the Declaration of Helsinki and was approved by the ethics committee of the University of Trier. Written informed consent was given by all participants. Participants were compensated with 10 €. All images and videos of people are used with the individuals’ informed consent to publish.

#### Setup

The study was conducted in the same laboratory as the first two studies. Again, all settings as well as the calibration procedure were identical to the other studies to ensure comparability of the results. For the evaluation of data quality, gaze data was recorded before and after the conversation (pre vs. post) as well as during the conversation itself.

##### Conversation partner

Participants interacted with one of two female RAs. For the conversation, as in experiment 2, the vertical partition wall was replaced by a chair and the RA took a seat. The seating position and other visual characteristics such as the background or conversation partner’s clothing were kept constant to the 3D condition of study 2. The faces of the two RAs involved in the study covered on average 8.1 × 5.8° or 18.6 × 13.3 cm, respectively.

#### Procedure

The testing sessions began with the identical calibration procedure as in study 1. After calibration and prior to the conversation, eye tracking quality was tested at four facial target locations (left eye, right eye, nose, mouth) of the conversation partner in randomized order (pre-conversation validation). Subsequently, a semi-structured conversation began. For this purpose, 12 questions of a published paradigm^43^ were alternately read out and answered by the participant and the RA. During the conversation the experimenter left the room and returned to test eye tracking data quality at the same facial target locations after the conversation (post-conversation validation).

#### Data analyses

##### Preprocessing

All calculations were based on averaged binocular data. For the validation sequences pre- and post-conversation outliers were excluded, as described in study 2. Of the 336 validation trials, markers of 28 trials (8.33%) had to be corrected due to outliers. One trial had to be excluded due to experimenter error (0.30%).

##### Definition of AOIs

AOIs were defined with the *limited-radius Voronoi tessellation method* using a radius of 2°, which was validated previously by Hessels and colleagues (2016). We added an ellipse around the face as an additional AOI to allow conclusions to be drawn about the total dwell time on the rest of the face (see Fig. 6a).

**Figure 5:**
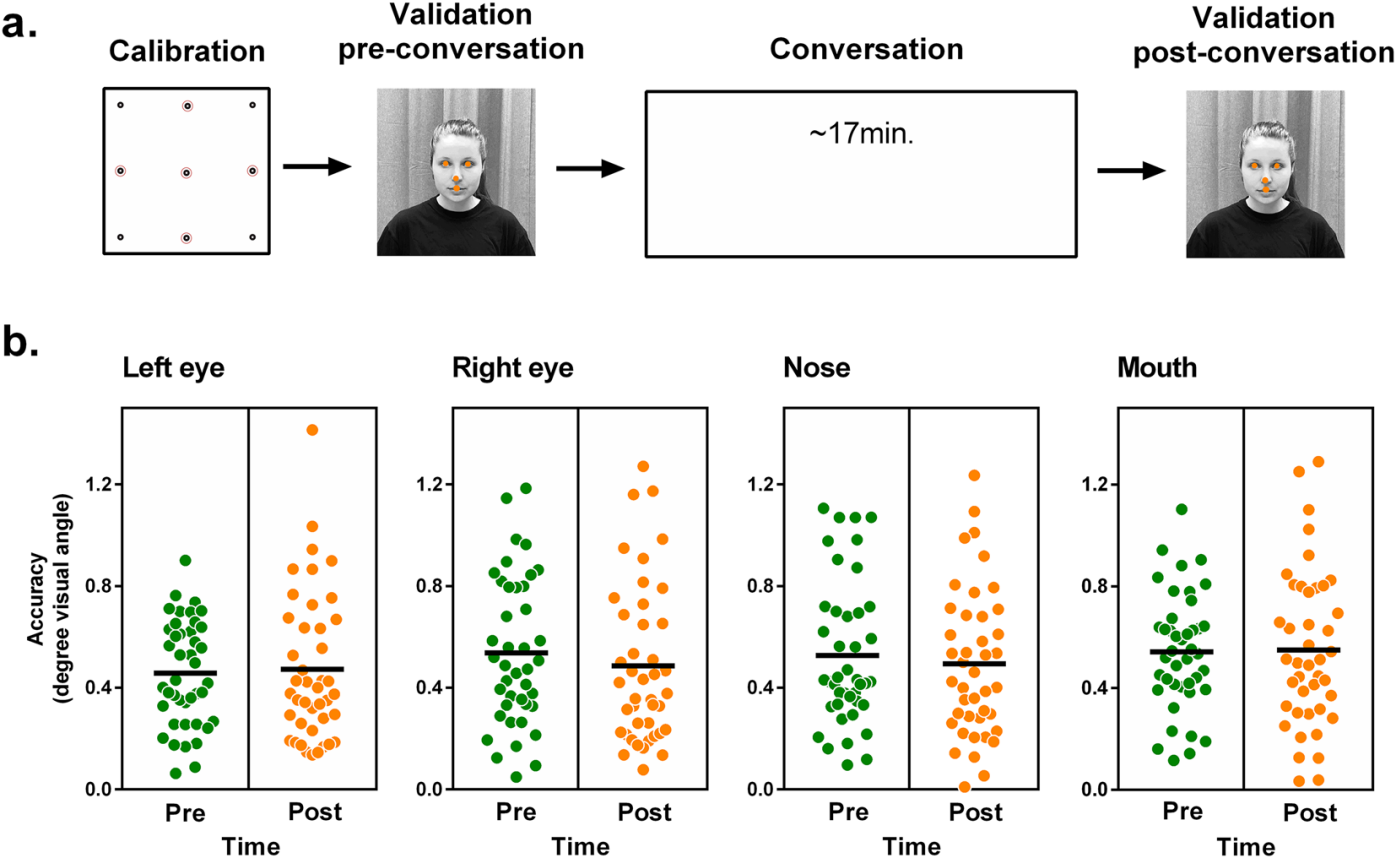
Accuracy for the validation sequence pre- and post-conversation. (**a**) The calibration was followed by a validation sequence on the face of the conversation partner. After a conversation of about 17 min, another validation sequence was added. (**b**) Accuracy for the different facial target locations in degrees visual angle. Black lines represent mean values. Lower values represent better accuracy.

##### Calculation of robustness, precision, and accuracy

Parameters of the validation sequences on the face of the conversation partner were calculated pre- and post conversation according to the 3D condition of study 2. Again, OpenFace was used for the automatic detection of the four facial target locations.

For the estimation of precision and accuracy during the conversation, a fixation algorithm according to the paper by Nyström and Holmqvist^44^ with a fixed peak velocity detection threshold of 15°/s was applied. For fixations in one of the predefined AOIs (eyes, nose, mouth), we assumed that the participants aimed at fixating the four target locations (center of the left and right pupil, nose tip and middle of the upper lip) that define the AOIs. Accuracy was estimated as the offset of the gaze data centroid from these target points, precision (SD) was calculated as the variance of the gaze data (as described in the "Methods" section of study 1).

In addition, movement of the conversation partner’s target locations due to naturally occurring facial expressions and head movements was quantified. As movement indices, the mean standard deviations (in degrees visual angle) of the four facial target locations detected through OpenFace were calculated. Precision (SD), accuracy and movement were calculated for listening and speaking phases of the participants separately. Movement indices were used to assess the influence of the conversation partner’s movements on data quality during these phases. For visualizing purposes we provide a video from one of our RAs during the conversation with a participant in the supplementary materials (see Supplementary Video S1).

##### Analysis of gaze pattern

For the conversation analysis, total dwell times of the segments were selected, in which the participants listened to the answers of their conversation partners. Total dwell time on a specific AOI was defined as the percentage of total frames within a specific segment comprising all gaze behavior (fixations, saccades, etc.). In addition, the percentage of missing data was calculated comprising all frames with no data available due to gaze outside the calibration area or loss of eye tracking.

##### Statistical analyses

Quality indices were compared pre- and post-conversation by using paired *t*-tests. Estimated precision and accuracy during the conversation, as well as the movement indices were set as dependent variables for two-way ANOVAs with the within-subject factors conversation phase (speak vs. listen) and target location (left eye, right eye, nose, and mouth). In cases of heterogeneity of covariance (Mauchly test of sphericity) Greenhouse–Geisser corrections were applied. The estimated precision and accuracy values during the conversation were then compared to the quality indices pre- and post-conversation using paired *t*-Tests. To evaluate the impact of movement on data quality Pearson correlations were calculated. All statistical analyses were conducted in R with the level of significance set to *p* < 0.05. Effect sizes are again reported as *r* for the *t*-tests and ƞ^2^_G_ for the ANOVAs.

### Results

On average, conversations lasted about 17 ± 3 min. Of these, participant spent on average ~ 34% (~ 6 min) of the time speaking and ~ 31% (~ 5 min) listening.

#### Robustness, precision and accuracy over the course of the experiment

Robustness, precision and accuracy were stable over the course of the experiment as indicated by non-significant comparisons for the indices before and after the conversation (see Table 3). The individual accuracy values of the four facial target locations during these validation sequences can be found in Fig. 5b.

**Table 3:** Robustness, precision and accuracy during the validation sequence before and after the face-to-face conversation. Quality indices were averaged for gaze data on left and right eyes, nose, and mouth.

	Pre-conversation		Post-conversation		*t*(41)	*p*	*r*
	Mean	SD	Mean	SD			
**Robustness**							
% valid data	95.54	6.90	93.96	11.17	0.98	0.332	0.15
**Precision (SD)**							
In degrees	0.45	0.16	0.45	0.18	− 0.09	0.932	0.01
In cm	1.03	0.39	1.03	0.41			
**Accuracy**							
In degrees	0.52	0.19	0.50	0.24	0.51	0.610	0.08
In cm	1.21	0.43	1.14	0.55			

Precision (SD) = standard deviation of data samples (Holmqvist et al., 2011).

The ANOVA on precision during the conversation with the within-subject factors conversation phase and target location revealed no significant main effect of conversation phase, *F*(1,41) = 0.16, *p* = 0.695, η^2^_G_ < 0.01, a significant main effect of target location, *F*(1.41,57.70) = 15.51, ε = 0.47, *p* < 0.001, η^2^_G_ = 0.10 and no significant interaction effect, *F*(2.13,87.40) = 0.54, ε = 0.71, *p* = 0.594, η^2^_G_ < 0.01.

The ANOVA on accuracy during the conversation with the same within-subject factors, revealed a significant main effect of conversation phase, *F*(1,41) = 4.81, *p* = 0.034, η^2^_G_ < 0.01, with slightly better accuracy during the speaking phases (see Table 4). Furthermore, a significant main effect of target location, *F*(2.09,85.76) = 9.86, ε = 0.70, *p* < 0.001, η^2^_G_ = 0.03, but no significant interaction effect, *F*(2.15,8.04) = 0.54, ε = 0.72, *p* = 0.596, η^2^_G_ < 0.01 was found.

**Table 4:** Precision, accuracy and movement during the face-to-face conversation. Values are reported in degrees visual angle (°) as a function of the participants’ speaking and listening phases.

	Speaking						Listening					
	Accuracy		Precision		Movement		Accuracy		Precision		Movement	
	Mean	SD	Mean	SD	Mean	SD	Mean	SD	Mean	SD	Mean	SD
Left eye	0.73	0.08	0.42	0.05	0.15	0.03	0.75	0.06	0.44	0.05	0.16	0.03
Right eye	0.69	0.08	0.35	0.02	0.32	0.04	0.71	0.07	0.35	0.03	0.31	0.03
Nose	0.69	0.07	0.34	0.02	0.31	0.03	0.71	0.07	0.34	0.02	0.32	0.04
Mouth	0.75	0.09	0.44	0.08	0.16	0.04	0.74	0.09	0.44	0.07	0.16	0.03

Precision = standard deviation of data samples (Holmqvist et al., 2011).

Finally, the ANOVA on movement of the partner during the conversation with the within-subject factors conversation phase and target location showed no significant main effect of conversation phase, *F*(1,41) = 2.11, *p* = 0.154, η^2^_G_ < 0.01, a significant main effect of target location, *F*(1.31,53.64) = 63.23, ε = 0.44, *p* < 0.001, η^2^_G_ = 0.46, and no significant interaction effect, *F*(1.98,81.27) = 0.66, ε = 0.66, *p* = 0.518, η^2^_G_ < 0.01.

Accuracy values obtained during the validation pre- and post-conversation were significantly better compared to the estimated accuracy values during the conversation, *t*(41) = 8.35, *p* < 0.001, *r* = 0.79. In contrast, precision (SD) values estimated during the conversation were slightly better compared to the value achieved pre and post conversation, *t*(41) = − 2.18, *p* = 0.035, *r* = 0.32.

#### Association of accuracy with movements of the conversation partner

The correlations of accuracy with the movement of different target locations (left eye, right eye, nose and mouth) in the face of the conversation partner, revealed no significant associations in the listening and speaking phases of the participant. Similarly, for precision (SD) non-significant associations were found, except for the movement of the nose in the speaking phase (*p* = 0.025 uncorrected), which did not survive correction for multiple testing. Correlation coefficients and scatterplots of these associations can be found in Supplementary Figs. S1 and S2.

#### Gaze pattern during conversation

**Figure 6:**
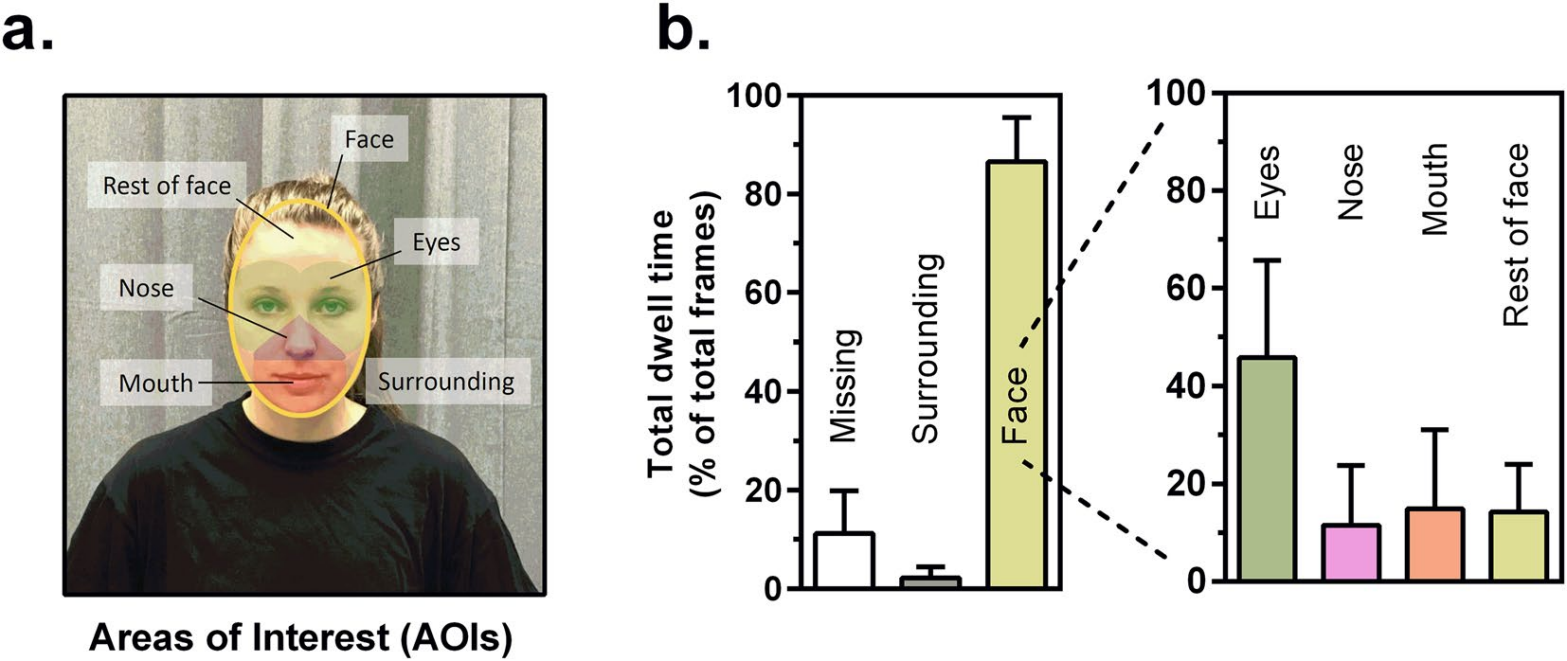
Total dwell time on different facial areas of interest (AOIs) during face-to-face conversation. (**a**) Location and size of AOIs. (**b**) Total dwell time for the face and specific facial areas. Bars represent mean ± s.e.

The analysis of total dwell time for the specific AOIs (see Fig. 6a) revealed that participants spent most of the time gazing at the face of the conversation partner (87%). Within the face area, the eyes attracted most of the attention (46%), while attention to the mouth, nose and the rest of face was evenly distributed at 10–15%. Approx. 3% of the time the participants looked at the surrounding, approx. 11% of the data was lost due to gazing outside the calibration area, or loss of tracking (see Fig. 6b).

### Discussion

The quality of eye tracking data recorded before and after a semi-standardized face-to-face conversation was comparable to study 1 and 2 and stable over the experiment. While study 2 indicated that the present setup allows measurement of gaze behavior on the face of a person with a typical viewing distance during face-to-face conversations and is robust against short interruptions, study 3 shows that the data quality is stable over a short conversation of approx. 15–20 min.

The accuracy estimated during the conversation was slightly lower than accuracy values calculated during the validation sequences. One likely explanation is the partly inaccurate inference of the true fixation point. Instead of announcing specific target locations during the conversation, we inferred the true fixation targets from the recorded data. For example, if the participant fixated within the AOI of the left eye, we assumed that the true fixation was intended on the center of the left eye pupil. However, we cannot rule out that the true fixation was elsewhere within the AOI, and therefore lower accuracy values reflect partly the inaccurate definition of true fixation targets rather than inaccurate eye tracking. In future studies, accuracy could be further investigated by specifying various features to be fixated on during a natural conversation with another person, although this would likely compromise the natural character of such an interaction.

In addition, the accuracy may have been further reduced by the naturally occurring delay between movement of the fixated stimulus and the observer’s gaze. In the validation sequences, the experimenter waited for the participant to look at the announced target, whereas this was not possible during the natural conversation. This could have lead to calculations of accuracy for frames in which the eyes were not fully adjusted to the altered position of the target, a situation that can be expected when the stimulus moves.

Nevertheless, the accuracy was uncorrelated to the movement index classified in speaking as well as listening phases during the conversation, indicating a low impact of stimulus movement on tracking accuracy in the current setup. In addition, this index suggests that movement during the natural conversation was generally sparse. However, we cannot rule out the possibility that higher degrees of stimulus movement (especially with high velocity) would result in lower tracking accuracy.

Importantly, regardless of the target movement, the accuracy and precision values (on average approx. 0.7 and < 0.5°, respectively) were sufficient to reliably distinguish gaze on different facial features at a typical distance during face-to-face conversations.

To explore gaze behavior during a face-to-face conversation, we applied an automatic AOI-generation method^45,46^ for facial features (eyes, nose, mouth) and analyzed total dwell times on these regions. A small proportion of approx. 10% of data was lost due to gaze outside the calibration area or lost tracking. The remaining data shows that the gaze behavior recorded with the present setup reveals a distribution of attention that is plausible for everyday face-to-face conversations. Participants' gaze was mainly directed to the face and within the face, especially to the eyes of their interaction partners. This pattern is consistent with findings of face exploration on static stimuli^47^, and in more naturalistic social encounters^4,48,49^. The investigation using dynamic stimuli has shown, amongst other things, that the fixation of the eye region is modulated by gaze behavior of the stimulus^20^ and the facial expression displayed^50^.

In sum, study 3 suggests that the setup described here is feasible for the recording of face-to-face conversations. Data quality appears to be sufficient, robust and largely independent of the participant’s and the conversation partner’s head movements and facial expressions occurring during a semi-structured conversation.

## General discussion

We have introduced an eye tracking setup that allows the measurement of gaze behavior in face-to-face conversations using a standard infrared remote eye tracker. We systematically evaluated eye tracking quality in this present scene-based setup by comparing the robustness, precision and accuracy with a standard display-based setup. We could show that scene-based eye tracking can be used to accurately measure gaze behavior on moving social stimuli. Visual attention on even small facial features such as the eyes in face-to-face conversations could be differentiated.

The main advantage of the present setup with remote eye trackers is the presumably reduced intrusiveness compared to mobile eye tracking devices such as eye tracker glasses^4^ or video-mediated conversations^26,30^. This might be particularly relevant for investigating eye contact in social interactions since the eye tracking device itself does not alter the salience of the eye region as the main target region of interest. Furthermore, the present study indicates that social interactions do not have to be mediated by video-based communication platforms to generate data for accurate differentiation of gaze allocations on facial features such as the eyes and the mouth. On the contrary, accuracy data with the real conversation partner and the analysis of gaze pattern during the conversation demonstrate that the scene-based setup can be applied in the study of social interaction e.g. a conversation between two people. Thus, this new setup could contribute to understanding the influence of natural contexts on socio-cognitive processes^51^.

Although the results of the current study are promising, it must be taken into account that the findings were generated using a specific remote eye tracker (Tobii X3-120) and have not yet been replicated with other devices. This is important to emphasize, as the quality of eye tracking varies depending on the model and manufacturer^34^. Furthermore, the current results were produced in the exact setup described in Fig. 1. Attention was paid to the thorough measurement of distances in the laboratory setup, and regular maintenance checks were carried out to verify that the distances and alignments remained unchanged.

Additionally, in the present studies the setup has only been validated in *single mode* using one eye tracker. It is however conceivable that the setup could be converted to *dual mode* for tracking two participants simultaneously while interacting with each other. A requirement for such an extension is the robustness and reliability of tracking after a short interruption, which could be shown in the present study (Study 2). This is necessary, because in dual mode, the participants have to be calibrated one after the other. Therefore, the partition wall with the calibration sheet must replace one participant’s chair while the other is being calibrated.

### Limitations

Some limitations should be mentioned. On the one hand, the display-based condition in the first study could have been improved if the 24 ” computer monitor (16:9) had been replaced by a monitor with a more uniform aspect ratio of e.g. 4:3 and a size that would have resulted in an identical calibration area with respect to the maximum gaze angle in both conditions. This would have led to a decreased maximum gaze angle and most likely to improved data quality at the edges of the display/calibration area. We refrained from doing so because the eye tracker has been tested under similar conditions using a 25 ” monitor with a 16:9 aspect ratio at the same viewing distance^52^ as a standard display-based setup. In fact, an accuracy of about 0.5° was comparable or slightly better than previously published with a maximum gaze angle at 30°^52^. On the other hand, the display-based setup had the purpose of serving as a control group for our scene-based setup and thus should produce eye tracking data under standard laboratory conditions (e.g. standard aspect ratio of screen, no use of chinrest, etc.).

Another limitation was the strict exclusion criteria used in all studies. We included participants with very little or no visual impairment. Therefore our results do not generalize to samples with a higher variance in eyesight. Future studies should address this issue by systematically evaluating eye tracking data quality measuring participants with visual impairments or with visual aids such as glasses or soft contact lenses.

Finally, usability in a face-to-face conversation was demonstrated for a semi-structured social interaction. It is possible that head movements or gazing outside the calibration area increase in less structured situations and the proportion of missing data could be larger while tracking accuracy could be lower. To counteract these potential limitations, future studies are needed that systematically investigate the influence of these context variables.

## Future directions

Since many interesting research questions on social interaction might be answered in real face-to-face interactions, follow-up studies need to validate the present setup in dual mode, using two eye trackers allowing the recording of eye movements of two participants while interacting. A possible application of this setup could be therapeutic sessions in which the gaze behavior of the patient during interaction with the therapist is recorded and might serve as a predictor for the quality of therapeutic alliance or therapy outcome. Moreover, the application of correction algorithms^31,53^ could further improve eye tracking data quality in 3D environments. Finally, a combination of eye tracking with unobtrusive motion tracking used in other setups^54^ could allow the assessment of non-verbal behavioral synchrony in social interactions.

## Conclusion

Eye tracking is a powerful tool to measure gaze behavior on facial features during face-to-face conversations and can be implemented with a standard infrared remote eye tracker. As long as crucial distances and angles are kept constant during a session and calibration is done with a standard procedure, data quality in terms of robustness, precision, accuracy and stability is comparable to well-established display-based eye tracking and sufficient to assess and quantify social gaze behavior such as eye contact. The present eye tracking setup can be used in various contexts of social interactions and has the potential to expand our knowledge on the role of visual attention in social interactions in healthy as well as clinical populations, such as autism spectrum and social phobia.

## Supplementary Information

Supplementary Information.

Supplementary Video 1.

## Data Availability

The datasets generated and analysed during the current studies are available in the Open Science Framework (OSF), https://osf.io/mnyvf/?view_only=d75223a2f21f4c66ac8e6b91fe3dec57.
