# Supplementary material for: Evaluation of an eye tracking setup for studying visual attention in face-to-face conversations
Source: Sci Rep. 2021 Jan 29;11:2661. doi: 10.1038/s41598-021-81987-x (PMC7846602; doi:10.1038/s41598-021-81987-x)
Supplement: Supplementary file 1 — Supplementary Information. [file 41598_2021_81987_MOESM1_ESM.pdf]

Evaluation of an eye tracking setup for studying visual attention in face-to-face conversations

Antonia Vehlen[1], Ines Spenthof[2], Daniel Tönsing[2], Markus Heinrichs[2], and Gregor Domes[1]

[1]Department of Biological and Clinical Psychology, University of Trier

[2]Department of Psychology, Laboratory for Biological and Personality Psychology, Albert-Ludwigs-University of Freiburg

Supplementary figure S1. Association of accuracy and movements of the conversation partner during speaking and listening of the participant in a face-to-face conversation. *S = participant speaking and L = participant listening. Uncorrected p-values.*

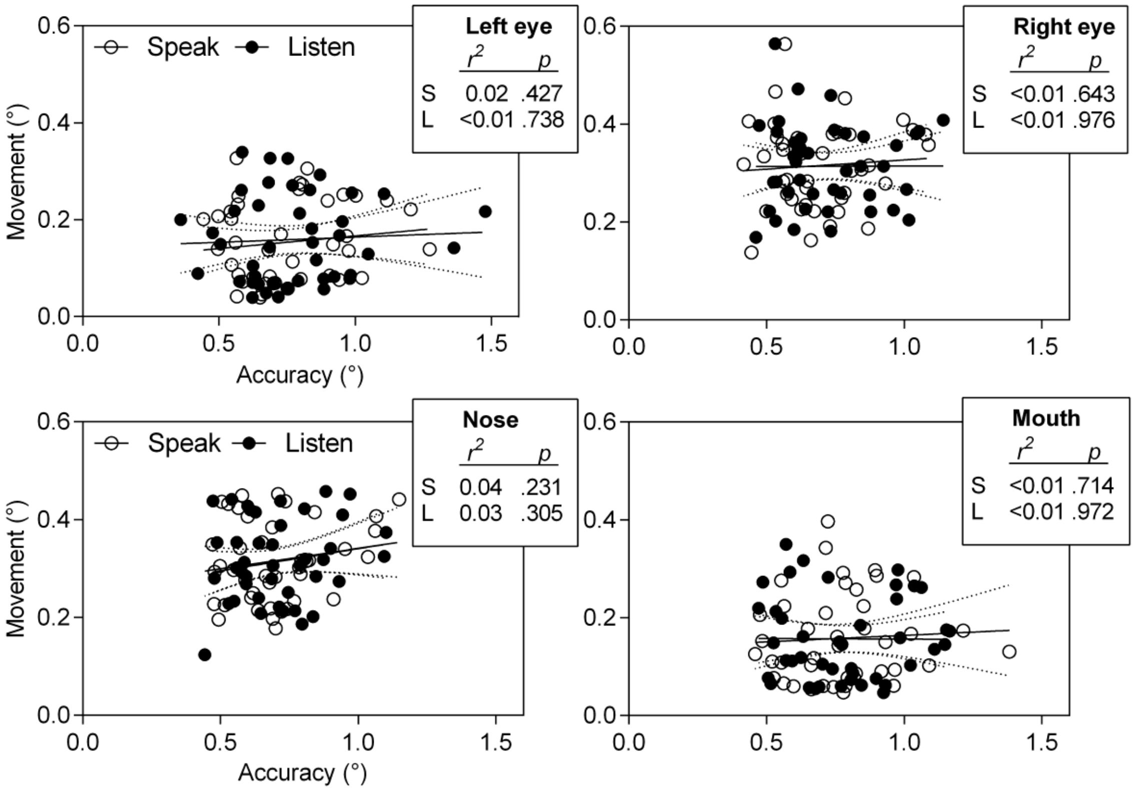

Supplementary figure S2. Association of precision and movements of the conversation partner during speaking and listening of the participant in a face-to-face conversation. *S = participant speaking and L = participant listening. Uncorrected p-values.*

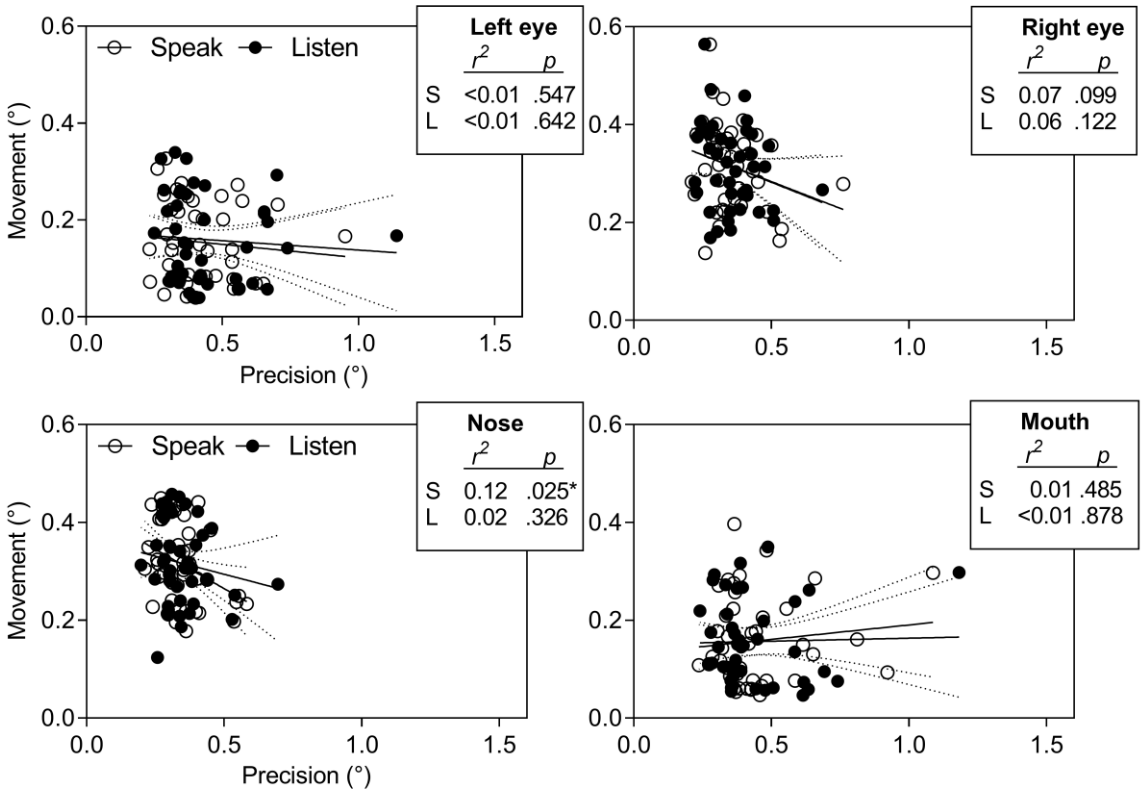

Supplementary video S1. A 10 sec sample sequence from a conversation of Study 3 showing the research assistant interacting with a participant (not shown). *The red dot visualizes the participants' gaze point, while the white dots correspond to the target points defining the AOIs.*

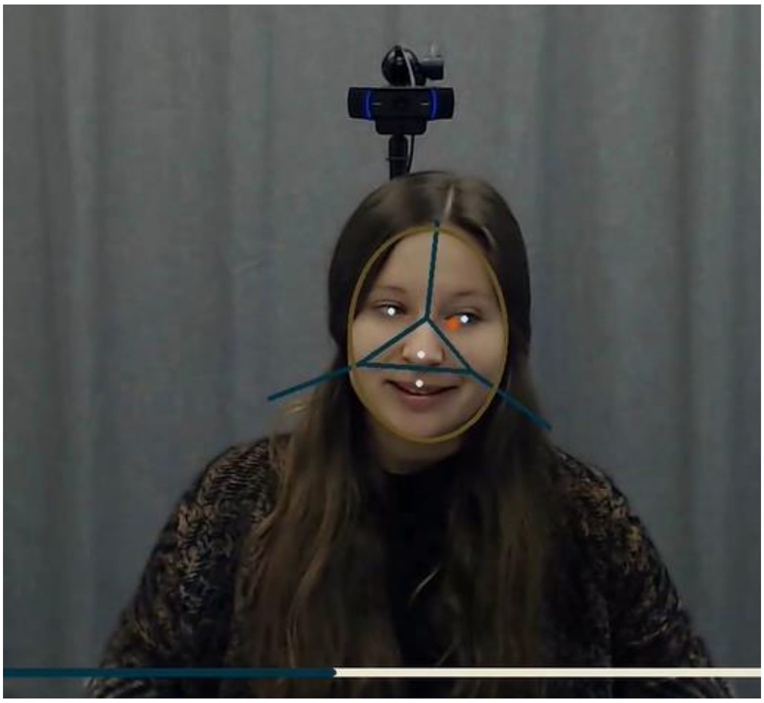

Screenshot
